# Root Foraging Influences Plant Growth Responses to Earthworm Foraging

**DOI:** 10.1371/journal.pone.0108873

**Published:** 2014-09-30

**Authors:** Erin K. Cameron, James F. Cahill, Erin M. Bayne

**Affiliations:** Department of Biological Sciences, University of Alberta, Edmonton, AB, Canada; Beijing Forestry University, China

## Abstract

Interactions among the foraging behaviours of co-occurring animal species can impact population and community dynamics; the consequences of interactions between plant and animal foraging behaviours have received less attention. In North American forests, invasions by European earthworms have led to substantial changes in plant community composition. Changes in leaf litter have been identified as a critical indirect mechanism driving earthworm impacts on plants. However, there has been limited examination of the direct effects of earthworm burrowing on plant growth. Here we show a novel second pathway exists, whereby earthworms (*Lumbricus terrestris* L.) impact plant root foraging. In a mini-rhizotron experiment, roots occurred more frequently in burrows and soil cracks than in the soil matrix. The roots of *Achillea millefolium* L. preferentially occupied earthworm burrows, where nutrient availability was presumably higher than in cracks due to earthworm excreta. In contrast, the roots of *Campanula rotundifolia* L. were less likely to occur in burrows. This shift in root behaviour was associated with a 30% decline in the overall biomass of *C. rotundifolia* when earthworms were present. Our results indicate earthworm impacts on plant foraging can occur indirectly via physical and chemical changes to the soil and directly via root consumption or abrasion and thus may be one factor influencing plant growth and community change following earthworm invasion. More generally, this work demonstrates the potential for interactions to occur between the foraging behaviours of plants and soil animals and emphasizes the importance of integrating behavioural understanding in foraging studies involving plants.

## Introduction

Foraging decisions involve integration of multiple environmental cues and are influenced by the presence of other organisms. The trade-offs made between resource acquisition and risk avoidance during foraging can thus be affected by the foraging decisions of other species, leading to complex interactions [1]. Such interactions among foraging behaviours are known to impact both population and community dynamics in animals [1], [2]. Though plants also integrate information in relation to foraging for soil resources [3] and show a trade-off between risk avoidance and resource acquisition [4], the potential for plant and animal foraging behaviours to interact has been rarely considered.

Root growth of plants is dynamic and is influenced by spatial and temporal heterogeneity in resource distributions [5]–[7]. Plants often place their roots preferentially in nutrient patches, although species vary in their ability to forage precisely (i.e., concentrate roots in nutrient patches) [8], [9]. Plants also alter their foraging in response to other types of environmental variation, such as the presence of mycorrhizal fungi [10] and herbivores [11], [12]. Moreover, recent work indicates that plants can integrate information about competitors and nutrient availability and adjust responses accordingly [3], [13].

Though it is broadly recognized that belowground interactions can affect aboveground community structure [14], the mechanisms through which detritivores (as opposed to root-feeding herbivores) influence plants are not well understood [15]. Both positive and negative effects of earthworms on plants have been reported, with positive impacts generally occurring in agricultural systems [15]. Less research has examined earthworms and plants in natural systems, but earthworm invasions in North American forests are causing dramatic shifts in plant community composition [16]–[19]. Native herbaceous plants have declined in richness and cover [16]–[18], while sedges and some non-native plants have increased following earthworm invasions into historically earthworm-free northern hardwood and boreal forests [17], [19]. Multiple possibilities have been proposed to explain these changes [20] including decreases in leaf litter thickness [16], changes in nutrient availability [20], disruption of mycorrhizal fungi associations [21], and consumption or movement of seeds [22]. Research has generally focused on indirect effects mediated through reductions in leaf litter thickness [16]–[18], [20]. In contrast, direct effects of earthworm burrowing have been largely overlooked as a factor influencing plant communities [15], with the exception of some research examining earthworm-seed interactions [22], [23].

Earthworm burrowing could affect plant foraging for soil resources in several ways: 1) indirectly via burrows acting as pathways for root elongation [24], [25]; 2) indirectly via altered nutrient distributions [15]; or 3) directly via consumption or abrasion [26], [27]. Burrows are often lined with earthworm excreta which have higher nutrient concentrations than surrounding soil, thereby creating nutrient patches [28], [29]. Although Darwin (1881) stated that earthworms “greatly facilitate the downward passage of roots of moderate size; and these will be nourished by the humus with which the burrows are lined” [30], surprisingly little attention has been paid to potential effects of earthworms on root growth and foraging behaviour.

We conducted a greenhouse experiment using two herbaceous perennial species native to the Canadian boreal forest: *Achillea millefolium* L. and *Campanula rotundifolia* L. *Campanula rotundifolia* has a low ability to forage precisely while *A. millefolium* displays a higher foraging precision [31]. We grew these plant species in the presence and absence of the deep-burrowing earthworm *Lumbricus terrestris* L., which is native to Europe but is an invader worldwide [32]. We predicted roots of both species would occur more frequently in burrows and cracks than in the soil matrix due to decreased soil resistance to root elongation in openings [33]. Greater occurrence of roots in burrows than cracks would suggest that increased nutrient availability from earthworm castings affected root distributions, while decreased occurrence would suggest roots were consumed or abraded by earthworms. We further predicted that *A. millefolium* roots would occur more frequently in burrows than cracks due to the ability of *A. millefolium* to forage precisely for nutrients, whereas *C. rotundifolia* roots would not display such a trend. As a result of this behaviour, biomass of *A. millefolium* was expected to be more positively affected by the presence of earthworms than *C. rotundifolia*, unless herbivory by *L. terrestris* was high.

## Materials and Methods

### Ethics Statement

No specific permits were required for the described field studies, as the location where samples were collected is not privately owned or protected. The field studies did not involve endangered or protected species. Data collected in this study will be available at https://era.library.ualberta.ca/public/home.

### Experimental Setup

**Figure 1:**
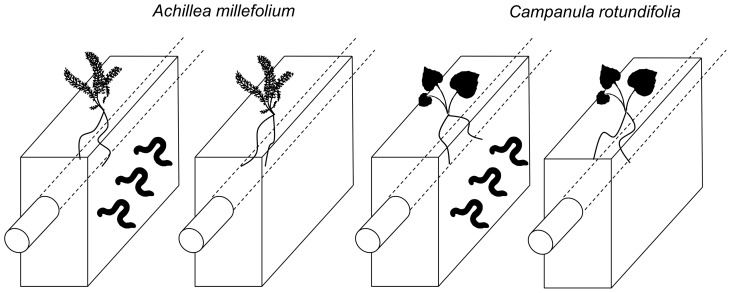
Schematic of experimental treatments. *Achillea millefolium* and *Campanula rotundifolia* were grown individually with and without earthworms in 15 replicate pots (27 cm × 11 cm × 26 cm). Three *Lumbricus terrestris* earthworms were added to each of the earthworm treatment pots. A transparent mini-rhizotron tube (5.7 cm in diameter) ran lengthwise approximately 5 cm below the soil surface of each pot to allow mini-rhizotron images to be obtained. Each mini-rhizotron tube ran through five adjacent pots.

To examine the effects of earthworm burrowing on root foraging, we grew *A. millefolium* and *C. rotundifolia* individually in pots with and without earthworms (Figure 1). Treatments were randomly assigned to pots, with 15 replicate pots per treatment. Pots were 27 cm × 11 cm × 26 cm deep wooden boxes filled with a mixture of mineral soil from the boreal forest of northeastern Alberta (54°36′N, 110° 59′W) and sand at a ratio of two parts soil to one part sand, with a uniform vertical soil structure. Soil was collected from an area where no earthworms were present. We mixed the soil with sand because sand reduces overall nutrient levels in the soil, makes it easier to extract roots, and reduces the development of gaps and cracks within the soil. Nonetheless, during filling of the pots, cracks formed naturally in the soil. These cracks changed very little in shape during the experiment and were thus distinguishable from burrows, which shifted slightly over time due to earthworm movements and were often lined with earthworm castings. A transparent acrylic tube (5.7 cm diameter) ran lengthwise through each pot approximately 5 cm below the surface of the soil to allow images to be taken with a mini-rhizotron camera (Bartz Technology). Each tube ran through a line of five pots. A mini-rhizotron camera was used to record root distributions monthly over 14 weeks (e.g., [3]).

Three adult *L. terrestris* individuals were added to each pot in the earthworm treatment. *Lumbricus terrestris* feeds on surface leaf litter but lives in permanent burrows that are typically vertical. Trembling aspen (*Populus tremuloides*) and balsam poplar (*Populus balsamifera*) leaves were supplied *ad libitum* as food, such that a thin layer of leaves (∼5 g) was continually present in pots from all treatments. Earthworms were added to the pots one week prior to the start of the experiment to allow burrow establishment before plants were added [34]. The rims of the pots were covered with a 1.5 cm thick strip of Velcro and a 10 cm tall strip of 1 mm plastic mesh was placed around the top edge of the pots to prevent earthworms from leaving the pots.

Seeds for *A. millefolium* and *C. rotundifolia* were obtained from Bedrock Seeds (Edmonton, Alberta) and Wild About Flowers (Calgary, Alberta), who collect and propagate seeds from local populations in Alberta. Following cold stratification, the seeds were planted in starter trays in late May and late June 2010, respectively. Seeds germinated in early to mid-July and were transplanted into experimental pots on 01-Sept-2010. We took images monthly starting on 1 September, for a total of four times over 14 weeks. The camera was positioned horizontally facing one side of the tube and images were taken along a belt transect in each tube. After 14 weeks, plants were harvested and shoots were dried at 60°C and weighed. Roots were stored at −20°C, washed in a 2 mm sieve, dried at 60°C, and weighed.

The experiment was conducted in a greenhouse in the Biological Sciences building at the University of Alberta. Pots were arranged in three blocks of 20, within which treatments were randomly assigned to the pots. Room temperature was maintained between 14.5 to 19°C and supplemental lighting was used to achieve a 16∶8 L:D light cycle. Plants were watered regularly to field capacity.

### Statistical Analyses

We digitized the locations of roots, burrows, and cracks in the soil in images obtained at four time steps (01 September 2010, 29 September 2010, 03 November 2010, 08 December 2010) in ArcGIS (v 10, Esri). Images from each pot (18 mm × 222 mm) were divided into 6 mm ×6 mm grid cells, with 111 cells per pot. We then determined occurrence of roots, burrows, and cracks within each cell at each time step.

Mixed effects logistic regression was used to examine root occurrence over time within grid cells containing burrows and cracks for each species separately. These models included void type (burrow, crack, or none), date, and the interaction of void type and date as fixed effects. The first date was not included in the analysis as planting had occurred just prior to imaging and there were no roots present at the depth of the mini-rhizotron tube. Pot identity and grid cell were used as random effects to account for correlations among grid cells within pots and within grid cells over time. We also performed post-hoc pairwise comparisons, with a Bonferroni correction for multiple testing, to examine root occurrence in cracks versus burrows at each time step. We focused our analysis on comparison of cracks versus burrows because detectability of roots is expected to be similar within these void types. In contrast, detectability might differ in the soil matrix versus in voids (cracks and burrows), as roots in voids are likely easier to see.

A similar mixed effects logistic regression analysis was used to examine differences in root mortality within grid cells containing burrows, cracks, and soil. We included a random effect to account for pot identity and a fixed effect to control for the date of initial colonization of cells by roots. In this analysis, we examined only grid cells with roots present during the experiment. Roots were considered to have died when they were no longer visible in the cell at subsequent time steps. Analyses were also performed using a random effect for tube identity (there were five pots along each tube), but they produced similar results and thus are not shown.

To assess effects of earthworms on root and shoot biomass, we used mixed effects linear regression with earthworm presence as a fixed effect and tube identity as a random effect. Species were analyzed separately. We also examined earthworm effects on biomass allocation to shoots versus roots using mixed effects linear regression with shoot biomass as the dependent variable and root biomass, earthworms, and the interaction between root biomass and earthworms as fixed effects. Tube identity was included as a random effect in this analysis as well. Normality was assessed by inspection of residuals and data were log transformed if non-normal. All analyses were conducted in Stata (v 12, StataCorp).

## Results

**Figure 2:**
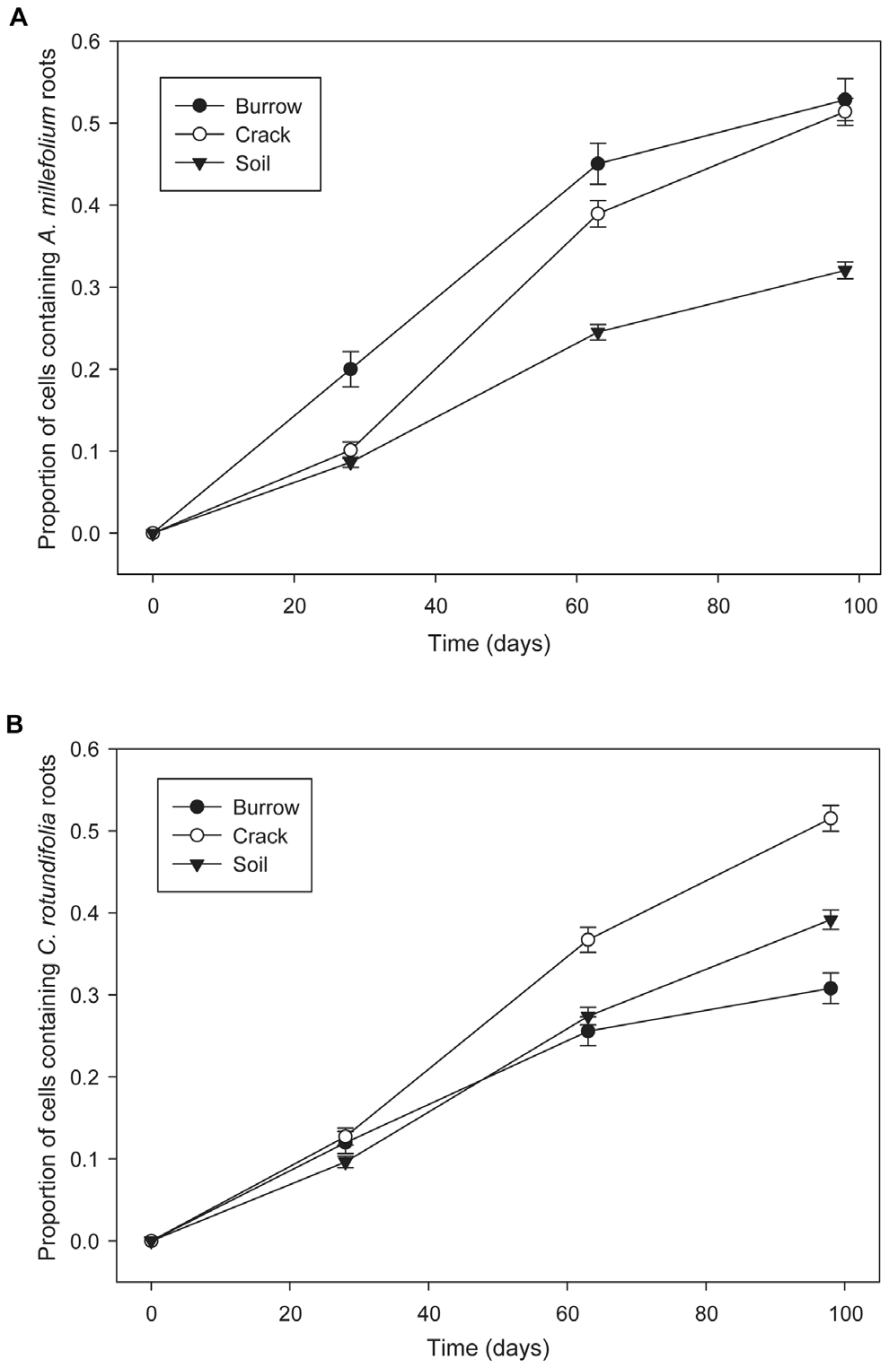
Occurrence of roots (±1 SE) in 6 mm × 6 mm grid cells containing burrows (filled circle), cracks (open circle), and soil (filled inverted triangle) for (A) *Achillea millefolium* and (B) *Campanula rotundifolia* over time.

The physical changes in soil structure caused by earthworm burrowing affected both plant species (Figure 2). Roots of *A. millefolium* (χ^2_2_^ = 85.84, P<0.0001) and *C. rotundifolia* (χ^2_2_^ = 10.37, P = 0.0056) more frequently occurred in voids than the soil matrix according to mixed effects logistic regression. This analysis alone cannot differentiate whether this pattern is due to increased detection probability (the camera can image ‘deeper’ into a void than into soil). Analyses contrasting root growth in burrows versus cracks do not have the potential confound of detectability differences, and thus are better suited to detecting root growth differences.

Only *A. millefolium* exhibited more extensive root growth in burrows than in soil cracks. There was a significant interaction between void type and time for *A. millefolium* (χ^2_4_^ = 29.80, P<0.0001). Post-hoc pairwise comparisons of root occurrence in cells with cracks versus burrows indicated that *A. millefolium* was initially more likely to place its roots in burrows (χ^2_1_^ = 19.38, P<0.0001), with roots occurring twice as frequently as in cracks (20% versus 10%). However, root occurrence in cracks became similar to burrows after two (χ^2_1_^ = 3.90, P = 0.14) and three months (χ^2_1_^ = 0.30, P = 0.99). In contrast, *C. rotundifolia* did not concentrate roots in burrows versus cracks at any point, although the distribution of its roots was also affected by an interaction between void type and time (χ^2_4_^ = 31.36, P<0.0001). Occurrence of *C. rotundifolia* roots was initially similar within cracks and burrows (χ^2_1_^ = 0.22, P = 0.99). After two months, roots were less likely to be present in burrows (χ^2_1_^ = 5.91, P = 0.045), and this difference became more pronounced by the end of the experiment with roots occurring 40% less frequently in burrows (χ^2_1_^ = 32.54, P<0.0001).

**Figure 3:**
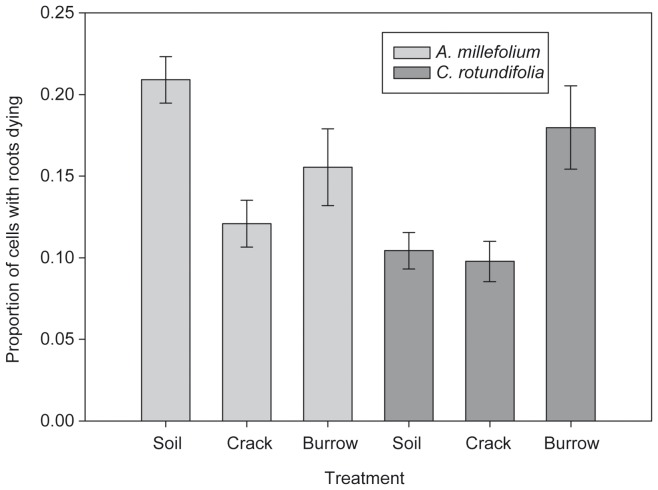
Proportion of 6 mm ×6 mm grid cells with roots dying (± SE), out of all cells occupied by roots during the experiment, in soil, cracks, and burrows for *Achillea millefolium* and *Campanula rotundifolia*.

For *C. rotundifolia*, the proportion of roots dying (no longer observed in a cell at subsequent time steps) in burrows was almost twice that in cracks and soil (Figure 3; χ^2_2_^ = 9.75, P = 0.0076). In contrast, *A. millefollium* roots experienced higher mortality in soil than voids (χ^2_2_^ = 20.56, P<0.0001), while mortality did not differ between cracks and burrows (χ^2_1_^ = 0.37, P = 0.54).

**Figure 4:**
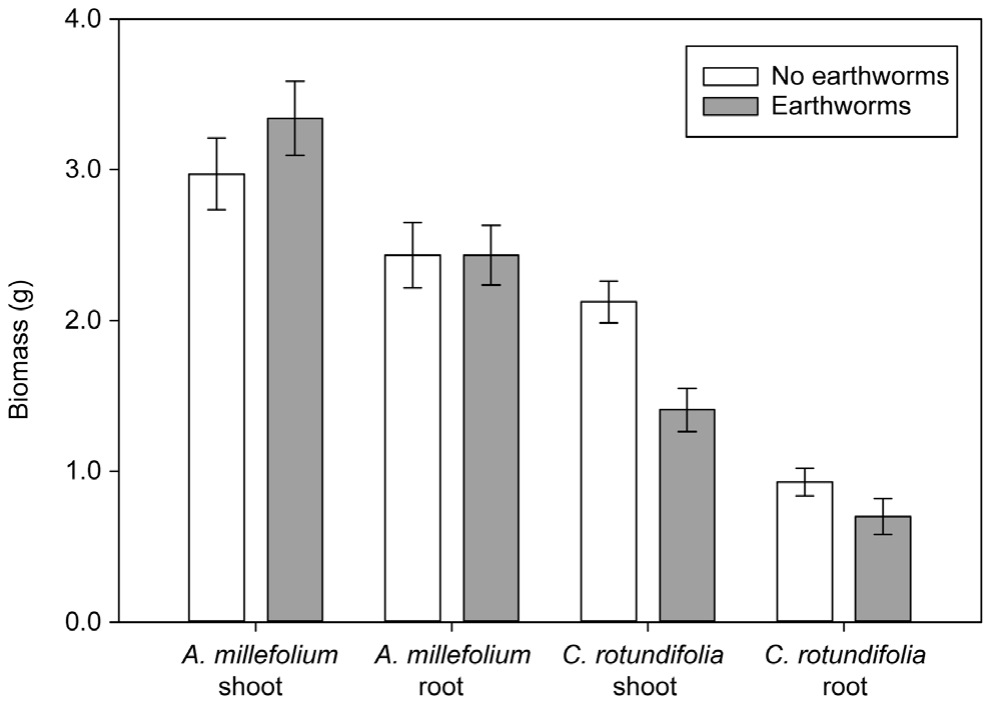
Shoot and root biomass in grams (± SE) for *Achillea millefolium* and *Campanula rotundifolia* with and without earthworms.

Despite the initial preference of *A. millefolium* roots for burrows, an overall growth response of *A. millefolium* to the presence of *L. terrestris* was not observed. Earthworms did not affect *A. millefolium* shoot biomass (Figure 4; F_1,28_ = 1.32, P = 0.25; general linear model) or root biomass (F_1,28_ = 0.31, P = 0.58). Similarly, earthworms did not influence allocation of biomass to shoots versus roots (F_1, 26_ = 1.38, P = 0.24) as indicated by a non-significant interaction effect of root biomass and earthworms on shoot biomass. However, consistent with our finding that *C. rotundifolia* roots occurred less frequently in burrows than soil or cracks at the end of the experiment, its overall biomass was lower when earthworms were present. Root and shoot biomasses were reduced by 25% and 33% respectively when earthworms were present (Root: F_1, 28_ = 6.04, P = 0.014; log-transformed data; Shoot: F_1, 28_ = 23.43, P<0.0001). Shoot to root ratio did not differ with earthworm presence (F_1, 26_ = 0.33, P = 0.56).

## Discussion

Both plant species responded to physical changes in soil structure, consistent with the idea that root growth is non-random. Roots occurred more frequently in cracks and burrows than in the soil matrix, where mechanical resistance to root growth is presumed to be higher [33], [35]. This suggests voids act as pathways for root elongation. Previous studies on earthworm burrowing in agricultural systems have generally found roots are more common in openings than in the soil matrix [25], [36], [37], although not always [38]. However, these studies were unable to distinguish whether burrows influenced root distributions or vice versa, or they did not differentiate between openings created by earthworms, soil pores, or other insects. By choosing an earthworm species that creates relatively permanent burrows and allowing burrow establishment prior to transplantation of seedlings, we were able to isolate the effects of burrowing on roots in our experiment, although detectability differences between voids and soil may have influenced comparisons of those locations.

Our results support the idea that earthworms influence plant root growth indirectly via nutrient redistribution. *Achillea millefolium* grew roots preferentially in burrows, where earthworm excreta lining the walls typically results in higher nutrient concentrations than in surrounding soil [28], [29]. *Campanula rotundifolia*, did not respond similarly and its roots were less likely to occur in burrows than cracks by the end of the experiment. In addition, the biomass of *C. rotundifolia* was significantly reduced in the presence of earthworms. Fine root biomass was similarly lower in forest plots invaded by earthworms than in earthworm-free plots in a study conducted in New York [39], but the species identities of roots were not determined. In our experiment, another factor that may have influenced plant biomass is that additional leaves, and consequently nutrients, were added to the pots with earthworms present in order to provide the earthworms with food. Although this amounted to only ∼2.5 g per week per pot, we could not add an equivalent amount of leaves to the earthworm-free control pots, as the seedlings were relatively small and would have been covered by the leaves. However, our results show the opposite pattern [8], [9] than would be expected if additional nutrients had affected plant biomass in the earthworm treatment, as biomass was not significantly higher for either species when earthworms were present. Competition between the plants and mycorrhizal fungi for nutrients in the litter may also have contributed to the net effects of earthworms on plant biomass and root foraging, but such competitive dynamics and their consequences for plants and fungi are poorly understood [40].

Consistent with a general understanding of the mechanisms that alter root proliferation [8], the observed patterns of root occurrence could be driven by differences in both root production and mortality rates. The lower occurrence of *C. rotundifolia* roots in burrows relative to cracks in our experiment could occur if *C. rotundifolia* reduced root production in burrows, or if roots in burrows were more likely to die. Initial root production of *C. rotundifolia* did not differ as a function of cracks versus burrows, though the proportion of roots dying in burrows was almost twice that in cracks. Thus we suggest this species did not alter root production, but instead suffered higher root mortality in burrows because of grazing or abrasion by earthworms. Earthworms have been shown to feed on roots [26], especially transparent rootlets [27]. However, it is unclear how frequently roots are consumed and whether particular plant traits influence root vulnerability to consumption by earthworms. For *A. millefolium*, we observed more new roots in burrows than cracks in the first month of growth, while mortality was highest in the soil matrix throughout the experiment. Mortality did not differ between the two void types. Combined, these results suggest *A. millefolium* altered both root production and mortality in response to the soil environment.

One explanation for the differing responses of *C. rotundifolia* and *A. millefolium* observed in our study is that the two species vary in their ability to concentrate their roots in nutrient patches (i.e., to forage precisely). Species that are high precision foragers should benefit from increased nutrient availability in earthworm burrows as *A. millefolium* did in our study, although such plants should experience greater costs if earthworms consume roots in burrows. However, we recognize that, as different species, *C. rotundifolia* and *A. millefolium* likely differ with respect to many other unmeasured variables that might influence their responses to soil pores and cracks. As a result, further investigation of additional species is needed to assess whether the behavioural activity of earthworms interacts with root foraging strategies to impact root distributions and plant growth. Little is known about the foraging strategies of plants in North American forests currently being invaded by earthworms. However, evidence indicates that invasive perennial forbs and grasses, a key group increasing in earthworm-invaded areas [19], have higher foraging precision than similar native species [41], [42]. Thus, differences in root foraging strategies in response to earthworm activity might account for some of the variability in population responses observed among plant species following earthworm invasion.

Our study provides evidence that the behavioural activity of earthworms can interact with root foraging to impact root distributions and plant growth. We examined the net effects of earthworms on root foraging, which combines both any gain due to fertilization (not measured) as well as any loss due to root feeding. Though not investigated here, root distributions have cascading effects on other ecosystem processes, including nutrient cycling [43], [44] and spatial structure of microbial populations [45]. Previous research indicates precisely foraging plant species can experience greater risks of root herbivory than less precise foragers due to increased herbivory in nutrient patches [11], [12]. Fine root biomass increased significantly when fertilizer and insecticide were applied, suggesting that herbivory costs are higher in more fertile microsites [11]. However, we did not observe a negative effect of earthworms on *A. millefolium* or greater mortality of its roots in burrows. The foraging plasticity of *A. millefolium* may allow it to compensate for any costs of consumption or abrasion, while species with lower ability to alter their foraging behaviour may experience only costs of earthworm burrowing.

Our results highlight the importance of considering foraging behaviour and interactions among species' foraging decisions when investigating plant foraging. Plants with more flexible foraging strategies may be less negatively impacted by earthworms, consistent with observations in animals where the ability to withstand novel stimuli is positively associated with behavioural plasticity (e.g., [46], [47]). Our experiment suggests earthworm effects on plant roots may be one factor involved in shifts in North American forest plant communities following earthworm invasions. However, additional investigation of the mechanisms involved in invasive earthworm effects on plant communities, and how effects vary depending on plant traits, is needed. More broadly, future research should examine how interactions between foraging strategies of plants and animals may influence plant populations and communities. Our study demonstrates soil animals influence root foraging via both direct and indirect pathways, leading to variable, although substantial, effects on plants.
